# Pathways to paediatric urology subspecialisation: a study of casemix, incumbent attitudes and opinions

**DOI:** 10.1007/s00345-023-04743-y

**Published:** 2024-01-13

**Authors:** F. O’Kelly, L. A. t’Hoen, B. Banuelos Marco, R. J. M. Lammers, S. Sforza, M. Hiess, E. Bindi, N. Baydilli, M. I. Donmez, I. Paraboschi, A. Atwa, A. F. Spinoit, B. Haid, S. Silay, The EAU-YAU Paediatric Urology Working Group

**Affiliations:** 1 Division of Paediatric Urology, Beacon Hospital, University College Dublin, Dublin, Ireland; 2 Department of Pediatric Urology, Erasmus MC University Medical Center, Rotterdam-Sophia Children’s Hospital, Rotterdam, The Netherlands; 3 Department of Urology, University Hospital El Clinico, Madrid, Spain; 4 Department of Urology, University Medical Center Groningen, Groningen, The Netherlands; 5 Paediatric Urology, Meyer Children Hospital, University of Florence, Florence, Italy; 6 Department of Pediatric Urology, Hospital of the Sisters of Charity Linz, Linz, Austria; 7 Department of Pediatric Surgery, AOU Delle Marche, Ospedale Pediatrico G Salesi, Ancona, Italy; 8 Department of Pediatric Urology, Erciyes University Faculty of Medicine, Kayseri, Turkey; 9 Division of Pediatric Urology, Department of Urology, İstanbul University İstanbul Faculty of Medicine, Istanbul, Turkey; 10 Department of Pediatric Urology, IRCCS Fondazione Ca Granda Ospedale Maggiore Policlinico, Milan, Italy; 11 Urology Department, Urology and Nephrology Center, Mansoura University, Mansoura, Egypt; 12 Department of Urology, Ghent University Hospital, Ghent University, Ghent, Belgium; 13 Department of Urology, Biruni University, Istanbul, Turkey

**Keywords:** Fellowship, Adolescent, Transitional, Training pathway, Workforce

## Abstract

**Objective:**

To identify any self-reported differences or attitudes towards certification, publication, or practice patterns between adult urology and paediatric general surgery-trained paediatric urology providers. There are no known published differences in clinical/operative/research outcomes in either group.

**Methods:**

An 18-item cross-sectional survey was compiled through the EAU Young Academic Urologists (YAU) office and disseminated to a trans-Atlantic convenience sample of current practising paediatric urologists. This was created using a mini-Delphi method to provide current semi-quantitative data relating to current opinions and attitudes of this cohort.

**Results:**

A total of 228 respondents completed the survey, with female respondents representing 37% and 34% for urology and paediatric general surgery, respectively. Nearly 90% overall respondents felt that a full 2-year paediatric fellowship program was very important and 94% endorsed a collaborative dedicated paediatric urology on call service, with 92% supporting the joint development of transitional care. Urology managed higher numbers of bedwetting (*p* = 0.04), bladder bowel dysfunction (*p* = 0.02), endourological procedures (*p* = 0.04), and robotics (*p* = 0.04). Paediatric general surgery managed higher numbers of laparoscopic reconstruction (*p* = 0.03), and posterior urethral valve ablation (*p* = 0.002).

**Conclusion:**

This study represents the first time that a cross-sectional cohort of paediatric urologists from different training backgrounds were compared to assess their productivity, practice patterns and attitudes. Paediatric urology is in a unique position to have two contributing specialities, with the ability to provide optimal transitional and lifelong care. We believe that there should be a strong emphasis on collaboration and to remove any historically-created barriers under policies of equity, diversity and inclusivity.

**Supplementary Information:**

The online version contains supplementary material available at 10.1007/s00345-023-04743-y.

## Introduction

Paediatric urology arguably begins with the study of childhood stone disease which was much more prevalent in the last two centuries than today. This may have been as a result of dehydration, diarrhoeal illness, or diets consisting of predominantly single grains [1]. The first childrens’ hospital to provide exclusive medical care to infants was the Hôpital des Enfants Malades in Paris in 1802. The Hospital for Sick Children on Great Ormond Street in London opened in 1852 largely due to the efforts of Charles West, with fund-raising assistance by Charles Dickens. The American Surgical Association was founded in 1879 by Samuel David Gross, followed nine years later by the American Pediatric Society in 1888. The Society’s 4th president William Osler remarked on surgical specialisation that “The rapid increase in knowledge has made concentration in work a necessity: specialism is here, and here to stay” [2]. Following the publication of the controversial Sheppard-Townsend Act in the USA, a large group of paediatricians broke away from the American Medical Association to form the American Academy of Pediatrics in 1931, from which a Section on Urology was established in 1960. The aim was to “improve the practice, expand the knowledge base, teach our successors… and disseminate paediatric genitourinary expertise to practitioners outside of our small domain. Our goal should be nuclei of paediatric urology centres and training programs in rigorous academic milieus, not only training our successors, but also teaching general urologists, paediatric surgeons, paediatricians and others” [3].

In contrast, the Royal College of Surgeons of England had only allowed the specialist designation of urology from general surgery in 1952, and paediatric surgery (whose progress correlated with advances in critical care medicine and anaesthesiology) had only begun to take-off as a speciality during the first world war. Having slowly conceded the treatment of orthopaedics, cardiac surgery and neurosurgery, pioneers like William Ladd, Robert Gross, and Orvar Swenson refused to concede paediatric urology. Disagreements arose between urologists and paediatric general surgeons as to who should operate on Wilm’s tumours in children and who had the best five-year survival rates [4]. Thus, a dichotomy of training pathways existed for paediatric urology with the Atlantic Ocean as a metaphorical “no-man’s land”. Depending on where one trained and became established, there would—in general—only be one route by which paediatric urology training pathways would be accepted.

Sub certification in paediatric urology took 25 years to come to fruition in North America and required leadership unanimity. Paediatric urology programmes were established to provided a platform and a framework for different groups to come together under a single sub-specialised umbrella. It was similar in concept to the justification cited by Sir Denis Browne in establishing the specialty of paediatric surgery when he said, “Paediatric surgery exists as a specialty, not to establish a monopoly but to establish a standard” [5–7].

There is, however, no known evidence for differences in clinical/operative outcomes between each camp, nor are there differences in the number and quality of publications produced. With the current trends towards gender/sex equality (50% of medical students in the USA are female, as are 46% of paediatric urology fellows in North American training, but only 9.9% of practising urologists) [8], diversity and inclusivity, as well as a push towards multidisciplinary input. It is argued that these siloes should be removed. The aim of this study was to assess if there were any noticeable differences in certification, casemix or publishing patterns between paediatric urology-fellowship trained urology and paediatric surgery-trained paediatric urologists, and to understand attitudes between each group in terms of fellowship training or appointment to consultant/attending level.

## Methods

An 18-item cross-sectional survey was compiled through the EAU Young Academic Urologists (YAU) office (Appendix 1) and disseminated to a trans-Atlantic convenience sample of current practising paediatric urologists. This Google Forms questionnaire was approved centrally by the YAU office and was created using a mini-Delphi method through the YAU research meetings to provide current semi-quantitative data relating to current opinions and attitudes of this cohort. Inclusion criteria for the target population were adult urologists who undertook regular paediatric work and general paediatric surgeons who considered themselves to be paediatric urologists. Exclusion criteria were those who did not undertake any paediatric urological work or did so infrequently. There were no interventions in this observational study. The survey was disseminated amongst the personal network of YAU members using email and social media focussing on highly active paediatric urologists.

The primary outcomes were to assess for evidence and duration of paediatric urology fellowship training, publishing practices and % time spent on a shortlist of index paediatric urological procedures and topics. Secondary outcomes included general case-mix and practice-types and attitudes to paediatric urology sub certification. Not all questions were answered by all respondents. There was an overall full completion rate of the survey of 93%. In those situations where questions were left unanswered, calculations were based on the those who did answer the question. Data were anonymised and analysed using GraphPad Prism 9.41 (USA; 2022). A p value of 0.05 was taken as significant. The study was deemed not to require ethics approval by the hospital research and ethics committee.

## Results

A total of 228 respondents completed the survey. Due to broad dissemination on specific social media channels, it was not possible to ascertain how many viewed or partially completed and then failed to submit the survey. As the survey was anonymised, it was also not feasible to cross-check answers against peer-reviewed publications. There was a 60:40% specialty split in favour of urology, with female respondents representing 37% and 34% for urology and paediatric general surgery respectively. There were no significant differences in respondent age, experience, practice-mix, or the availability of a paediatric urology fellowship in their own institution in either group. 29% respondents undertook a paediatric urology fellowship in North America, 60% undertook a fellowship in Europe and 11% subspecialised in Australia/Asia/South America.

Those respondents who initially trained through adult urology were statistically more likely to have a higher sole commitment to paediatric urology than those who trained in paediatric generally surgery (> 80% paediatric urology 83 vs. 56%, *p* = 0.0001) and were more likely to provide dedicated out of hours emergency cover in paediatric urology (96 vs 80%, *p* = 0.002). Respondents who initially trained in adult urology were also statistically cumulatively more likely to have completed longer fellowships in paediatric urology (2 + years, 60 vs 0.40%, *p* = 0.006)) than those who trained in paediatric general surgery (*p* = 0.02) and were more likely to publish more peer-reviewed manuscripts (5+) per annum (12 vs 3%, *p* = 0.02). Both groups felt that a paediatric urology fellowship was of very high importance (Table 1).

**Table 1:** Clinical and Occupational Characteristics between Urologists and Paediatric General Surgeons practising Paediatric Urology

Comparator	Urological surgery	Paediatric general surgery	*p* value
No. of respondents	137	91	
Of which female (%)	37.5	34.1	0.75
Age			
< 45 years	54	41	
> 45 years	36	47	0.1
Attending experience (%)			
< 5 years	32	25	
6–10 years	21	25	
11–20 years	17	18	
20 + years	18	17	
% Paediatric urology commitment			
< 80	17	44	
80 +	83	56	**0.0001**
Practice type			
Academic	75	73	
Non-academic	13	12	1
Public only	51	54	
Private only	15	7	0.1
% Exclusive paediatric urology out of hours cover	96.6	80	**0.002**
Length of paediatric urology fellowship			
0–1 year	10	24	0.07
1–2 years	18	21	0.59
2 + years	60	40	**0.006**
Peer-reviewed publications (last 5 years)			
0–2 per annum	49	59	0.13
3–4 per annum	36	28	0.88
5 + per annum	12	3	**0.02**
Available paediatric urology fellowship at own institution (%)	55	47	
Perceived importance of dedicated paediatric urology fellowship (Linear Scale 1–10)			
Mean	9	8	
Median	10	8	
Range	5–10	3–10	

Bold values indicate statistical significance

**Fig. 1:**
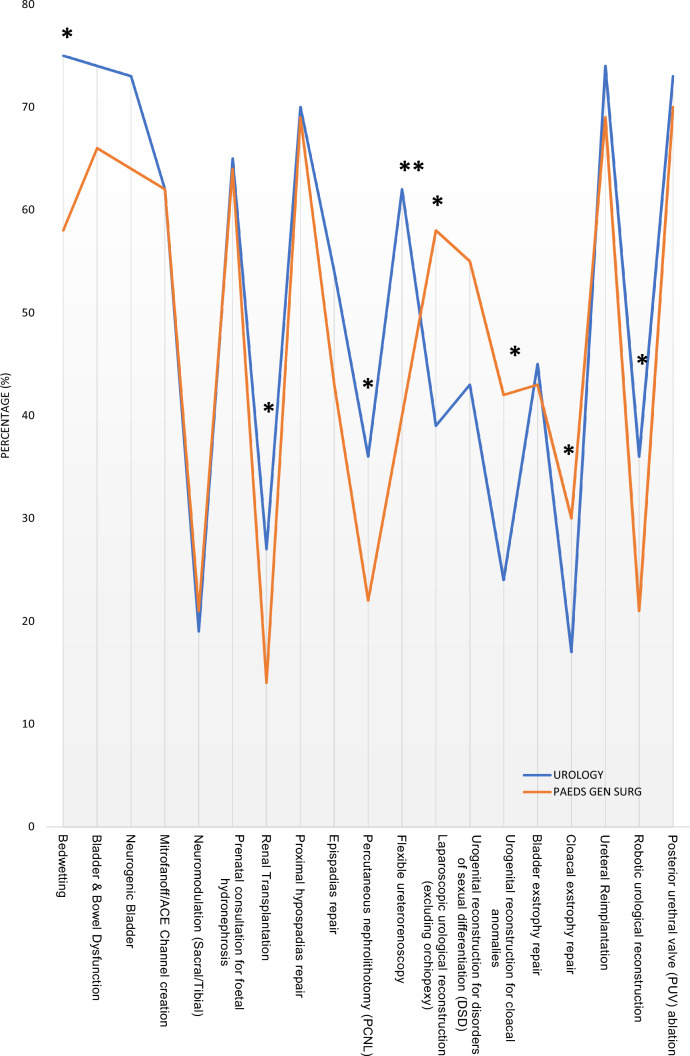
Clinical and operative casemix of paediatric urology respondents broken down by initial training pathway (adult urology and paediatric general surgery). The *Y*-axis indicates the percentage of paediatric urologists actively and regularly engaged in those listed cases (*X*-axis) (*: *p* < 0.05; **: *p* < 0.01)

Respondents were also asked about clinical and operative casemix in their practice across a number of pre-determined conditions through the mini-Delphi consensus. The results illustrated that there were no differences in those who dealt with the clinical management of bladder/bowel dysfunction, neurogenic bladder, prenatal hydronephrosis, or disorders/differences in sexual development (DSD). There were also no statistically significant differences in the operative management of Mitrofanoff/Antegrade Continence Enema channel creation, hypospadias, epispadias, ureteral reimplantation, or posterior urethral valve ablation. Those who initially trained in adult urology were more likely to manage bedwetting (*p* = 0.02), and to perform renal transplantation (*p* = 0.05), percutaneous nephrolithotomy (*p* = 0.05), flexible ureterorenoscopy (*p* = 0.004) and robotic-assisted reconstructive surgery (*p* = 0.03). Respondents who had initially trained in paediatric general surgery were more likely to perform laparoscopic-assisted reconstructive surgery (*p* = 0.02) and to be involved with the repair of cloacal anomalies (*p* = 0.01) and cloacal exstrophy (*p* = 0.05) (Fig. 1).

**Fig. 2:**
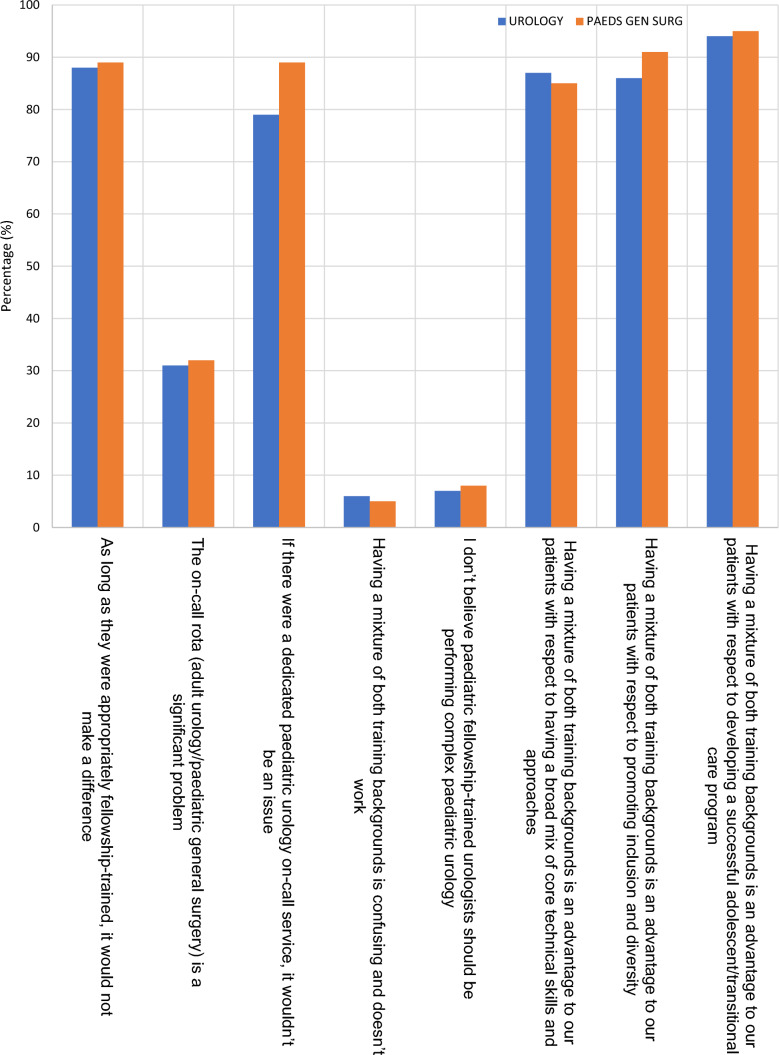
Attitudes and opinions of paediatric urology respondents broken down by initial training pathway (adult urology and paediatric general surgery). The *Y*-axis indicates the percentage sentiment of paediatric urologists towards those listed opinions (*X*-axis)

Respondents were finally asked to express their views regarding eight statements relating to the provision of paediatric urology specialist care. There were no statistically significant differences in responses to the statements between groups. Nearly 90% respondents felt that there was no particular difference whether paediatric urology services were provided by those who initially trained in either adult urology or paediatric general surgery as long as the attending/consultant was appropriately fellowship trained. Similar numbers of respondents (> 80%) also felt that having a mixture of backgrounds was advantageous to patients due to the differences in skill mix and in promoting inclusion and diversity. Greater than 90% respondents felt that having a mixture of training backgrounds was advantageous to patients with respect to developing a successful adolescent/transitional care program. Nearly one third respondents felt that out-of-hours cross cover was challenging, however > 80% felt that if there was a dedicated paediatric urology out-of-hours/emergency rota that this wouldn’t be an issue. Less than 10% respondents believed that having a mixture of training backgrounds was confusing and wouldn’t work and that paediatric fellowship-trained urologists should not be performed complex paediatric urology (Fig. 2).

## Discussion

The study has demonstrated a number of similarities and differences between paediatric urologists trained in adult urology and those trained in paediatric general surgery. The demographics and practice types were broadly similar between both groups. It is unclear as to why adult urologists tended to have longer fellowships in paediatric urology. Arguably this may be down to the fact that they are not as used to handling smaller tissue, or may be reflective of the requirements and expectations of the countries that they train and work in. Similarly, those institutions which have traditionally employed paediatric general surgeons may not necessarily require more than one year of fellowship, however the European Society of Paediatric Urology state a minimum training requirement of two years in paediatric urology (https://www.espu.org/images/ebpu/ETR_Paediatric_Urorolgy_2020_05_04_v2.pdf). There is no available literature around these differences. It is also likely that the increased dedication of adult-trained paediatric urologists dealing exclusively with paediatric urology for on-call rota also reflects differences in work commitments amongst hospitals where paediatric general surgeons may be required to provide emergency out-of-hours cover for general surgical conditions. This has led to significant trainee attrition over the last number of years with figures as high as 4.2% which in turn affects the ability to sub-specialise care [9, 10].

The case mix for adult urologists and paediatric general surgeons demonstrated significant overlap, however there were subtle differences in operations which may reflect background training. The adult-trained paediatric urologists were more likely to perform endourological procedures and robotic-assisted procedures, whereas the paediatric general surgeons were more likely to perform laparoscopic-assisted procedures and those involving hindgut reconstruction. There has been a general narrowing in paediatric general surgery casemix over the last decade towards a more narrow focus of practice which may also be reflected in this [11]. Other evidence points towards a proportional increase in procedures of lesser complexity compared to prior decades with lower volume higher complexity procedures being referred to supra-specialised centres [12, 13].

Despite these differences, the majority of respondents were unanimous in their opinions regarding pathways of training. Both urologists and paediatric surgeons believed a fellowship to be essential and broadly welcomed collaboration between both groups as they felt that it had the ability to enhance patient care, especially in the area of adolescent/transitional care. Given the concerns of paediatric urology fellows regarding job availability and the financial pressures associated with fellowship training, having an integrated approach would allow for a network effect to increase patient services, expand and enhance departments and would go some way towards reducing some of these concerns [14].

Those few countries who have traditionally kept these pathways separate (UK, Ireland) for those pursuing a career solely in paediatric urology are now under pressure to manage faculty appointment to allow for a greater access to trainee education and in the light of consultant/attending shortages. It would appear superficially intuitive that having well-trained paediatric urologists is a given in developing an integrative model of care, yet this has not to our knowledge ever been demonstrated in the literature.

This study was—as all survey studies are—limited by a certain risk of inclusion bias and an unknown number of not answered questionnaires for unknown reasons. However, with a non-directed dissemination strategy yielding 40%/60% inclusion of both specialties and with a relatively large number of respondents, we believe that the results presented are representative. Furthermore, one cannot ensure a geographically-balanced reply from different countries, in who’s individual circumstances may influence the results of such a survey. Given the findings of this study, we would strongly endorse that an integrative and collaborative approach be adopted worldwide to allow for the optimal management of these patients and in countries with both training backgrounds available, forming departments with attendings/consultants from both specialties might be an appealing option instead of fostering competition.

## Conclusion

This study represents the first time that a cross-sectional cohort of paediatric urologists from different training backgrounds were compared to assess their casemix, practice patterns and attitudes. Paediatric urology is in a unique position to have two specialities in the supply chain, each adding complementary competences and nuances to the large spectrum of clinical practice. Furthermore, providing optimal transitional and lifelong care is an asset valuable to many patients. Historical barriers to practising as a paediatric urologist have no role in the modern context, and any artificial silos should be scrutinised under policies of equity, diversity and inclusivity.

## Supplementary Information

Below is the link to the electronic supplementary material.

Supplementary file1 (DOCX 37 KB)

## Data Availability

Data is available on request.

## References

[1] Acar B, Inci Arikan F, Emeksiz S, Dallar Y (2008) Risk factors for nephrolithiasis in children. World J Urol 26(6):627–630 18810456 10.1007/s00345-008-0331-7

[2] Hinman FH Jr (1991) American pediatric urology. Norman Publishing, San Francisco p, p 21

[3] Bloom DA (2003) State-of-the Section Address: American Academy of Pediatrics, Section on Urology. The origin of a species: pediatric urology. J Urol 170(4 Pt 2):1488–1492 14501642 10.1097/01.ju.0000091213.24372.01

[4] Innes Williams D (2003) The history of paediatric urology: personal recollections 1948–1978. BJU Int 92(1):1–3 12968999 10.1046/j.1464-410x.92.s1.7.x

[5] Rushton HG, Kaplan GW, Mitchell ME, Caldamone AA (2019) Road to pediatric urology subcertification: a 25-year journey. J Pediatr Urol 15(2):108–111 30967357 10.1016/j.jpurol.2019.02.017

[6] Ritchey ML (2012) Pediatric urology: a “grown-up” subspecialty. J Urol 187(1):7–8 22088347 10.1016/j.juro.2011.10.050

[7] Williams DI (1999) Denis Browne and the specialization of paediatric surgery. J Med Biogr 7(3):145–150 11623911 10.1177/096777209900700304

[8] Haslam RE, Collins A, Martin LH, Bassale S, Chen Y, Seideman CA (2021) Perceptions of gender equity in pediatric urology. J Pediatr Urol 17(3):406.e1-406.e7 33558178 10.1016/j.jpurol.2021.01.011

[9] Khalil K, Sooriyamoorthy T, Ellis R (2022) Retention of surgical trainees in England. Surgeon. 10.1016/j.surge.2022.09.001. (**Advance Online Publication**) 36163150 10.1016/j.surge.2022.09.001

[10] Shapiro E, Cooper CS, Greenfield S, American Academy of Pediatrics Section on Urology (2017) The American Academy of Pediatrics Workforce Survey for the Section on Urology 2015. J Pediatr Urol 13(1):68–72 28089294 10.1016/j.jpurol.2016.09.002

[11] Reich DA, Herbst KW, Campbell BT (2019) The recent evolution of the breadth of practice for pediatric surgeons in the United States, 2005–2014. Pediatr Surg Int 35(4):517–522 30607543 10.1007/s00383-018-04433-6

[12] Bruns NE, Shah MA, Dorsey AN, Ponsky TA, Soldes OS (2016) Pediatric surgery—a changing field: national trends in pediatric surgical practice. J Pediatr Surg 51(6):1034–1038 26987709 10.1016/j.jpedsurg.2016.02.079

[13] Malangoni MA, Biester TW, Jones AT, Klingensmith ME, Lewis FR Jr (2013) Operative experience of surgery residents: trends and challenges. J Surg Educ 70(6):783–788 24209656 10.1016/j.jsurg.2013.09.015

[14] Husmann DA, Routh JC, Hagerty JA, Cannon GM, Gomez P, Cheng EY, Skoog S (2011) Evaluation of the United States pediatric urology workforce and fellowships: a series of surveys performed in 2006–2010. J Pediatr Urol 7(4):446–453 21324750 10.1016/j.jpurol.2010.12.009

