# Supplementary material for: Pathways to paediatric urology subspecialisation: a study of casemix, incumbent attitudes and opinions
Source: World J Urol. 2024 Jan 13;42(1):34. doi: 10.1007/s00345-023-04743-y (PMC10787691; doi:10.1007/s00345-023-04743-y)
Supplement: Supplementary file 1 — Supplementary file1 (DOCX 37 KB) [file 345_2023_4743_MOESM1_ESM.docx]

|  | The Global Pathway to Paediatric Urology - Urology vs Paediatric General Surgery |
| --- | --- |
|  |  |

**Prior to embarking upon subspecialty training in paediatric urology, what did you complete your residency in?**

Paediatric General Surgery

Adult Urology

#### In what country did you do your residency/specialty training?

#### In what country do you currently work?

#### What is your age?

18-24

25-34

35-44

45-54

55-64

65+

Prefer not to say

#### What is your gender?

Male

Female

Prefer not to say

#### For those who have completed training, how long have you been practicing as a paediatric urologist?

<5 Years

5-10 Years

10-20 Years

>20 Years

#### Typically, what percentage (%) of the week would you spend practising paediatric urology

<50%

50-80%

>80%

I don't currently practice any paediatric urology

**What *best* describes your practice type in paediatric urology?**

Academic Public

Academic Private

Non-Academic Public

Non-Academic Private

Academic Mixed Public/Private

Non-Academic Mixed Public/Private

#### If providing out of hours on-call services, do you have a dedicated paediatric urology rota?

Yes

No

I do not currently have on-call commitments

#### Did you undertake a dedicated paediatric urology fellowship, and if so, how long was it?

<1 Year

1-2 Years

2 Years

>2 Years

I did not undertake a dedicated paediatric *urology* fellowship

**As part of your fellowship, did you undertake a non-clinical/research year?**

Yes

No

N/A

#### In what country/countries did you undertake your fellowship training?

**As part of your fellowship, which on-call out of hours rota did you cover (Tick all that apply)?**

Paediatric General Surgery Only

Paediatric Urology Only

Both Paediatric General Surgery and Urology

Adult Urology

#### In your own practice do you personally manage/perform the following? (Check all that apply)

Bedwetting

Bladder & Bowel Dysfunction

Neurogenic Bladder

Mitrofanoff/ACE Channel creation

Neuromodulation (Sacral/Tibial)

*Prenatal* consultation for foetal hydronephrosis

Renal Transplantation

Proximal hypospadias repair

Epispadias repair

Percutaneous nephrolithotomy (PCNL)

Flexible ureterorenoscopy

Laparoscopic urological reconstruction (*excluding orchiopexy*)

Urogenital reconstruction for disorders of sexual differentiation (DSD)

Urogenital reconstruction for cloacal anomalies

Bladder exstrophy repair

Cloacal exstrophy repair

Ureteral Reimplantation

Robotic urological reconstruction

Posterior urethral valve (PUV) ablation

#### How many peer reviewed papers have you published in the last 5 years?

0-5

5-10

#### 10-15

#### 15-20

#### >20

#### How important is a dedicated fellowship in paediatric urology in your personal opinion? (0=irrelevant; 10=extremely important)

#### Do you have an active paediatric urology fellowship program in your own institution?

Yes

No

**Post-paediatric urology fellowship training, what are the factors that would influence appointment of one group or another to your institution *(Please tick all that apply)*?**

As long as they were appropriately fellowship-trained, it would not make a difference

The on-call rota (adult urology/paediatric general surgery) is a significant problem

If there were a dedicated paediatric urology on-call service, it wouldn’t be an issue

Having a mixture of both training backgrounds is confusing and doesn’t work

I don’t believe paediatric fellowship-trained urologists should be performing complex paediatric urology

Having a mixture of both training backgrounds is an advantage to our patients with respect to having a broad mix of core technical skills and approaches

Having a mixture of both training backgrounds is an advantage to our patients with respect to promoting inclusion and diversity

Having a mixture of both training backgrounds is an advantage to our patients with respect to developing a successful adolescent/transitional care program
